# Clinical characteristics and prognoses in pediatric neuroblastoma with bone or liver metastasis: data from the SEER 2010–2019

**DOI:** 10.1186/s12887-024-04570-z

**Published:** 2024-03-07

**Authors:** Xudong Zhao, Zhuofan Xu, Xiaochuan Feng

**Affiliations:** Department of Pediatric Surgery, Zhongshan City People’s Hospital, No. 2 Sunwen East Road, Guangdong Zhongshan, 528400 People’s Republic of China

**Keywords:** Neuroblastoma, Bone metastasis, Liver metastasis, Clinical characteristics, Survival, Treatment

## Abstract

**Background:**

To investigate clinical characteristics, prognoses, and impacts of treatments on prognoses of neuroblastoma patients with bone or liver metastasis.

**Methods:**

This retrospective cohort study extracted data from the Surveillance, Epidemiology, and End Results (SEER) database 2010–2019. The outcomes were 3-year cancer-specific survival (CSS) and 5-year CSS. Multivariable COX risk proportional models were established to assess the association between metastasis types and CSS. Hazard ratios (HRs) and 95% confidence intervals (CIs) were estimated.

**Results:**

Totally 425 patients with metastatic neuroblastoma were eligible for 3-year CSS analysis and 320 for 5-year CSS analysis. For 3-year follow-up, 62 (14.59%) patients had liver metastasis alone, 289 (0.68%) had bone metastasis alone, and 74 (17.41%) had both liver and bone metastasis. For 5-year follow-up, 44 (13.75%) patients had liver metastasis alone, 223 (69.69%) had bone metastasis alone, and 53 (16.56%) had both liver and bone metastasis. Significant differences were observed in age, tumor size, surgery for the primary site, chemotherapy, radiation, brain metastasis, lung metastasis, and vital status between patients with liver metastasis alone, bone metastasis alone, and both liver and bone metastasis (all *P* < 0.05). Compared with patients with liver metastasis alone, patients with bone metastasis alone (HR = 2.30, 95%CI: 1.10–4.82, *P* = 0.028) or both (HR = 2.35, 95%CI: 1.06–5.20, *P* = 0.035) had significantly poorer 3-year CSS; patients with bone metastasis alone (HR = 2.32, 95%CI: 1.14–4.70, *P* = 0.020) or both liver and bone metastasis (HR = 2.33, 95%CI: 1.07–5.07, *P* = 0.032) exhibited significantly worse 5-year CSS than those with liver metastasis alone. In patients with bone metastasis, those with chemotherapy had significantly better 3-year CSS than those without (HR = 0.24, 95%CI: 0.07–0.75, *P* = 0.014). Among patients with liver metastasis, receiving radiation was associated with significantly worse 3-year CSS (HR = 2.00, 95%CI: 1.05–3.81, *P* = 0.035).

**Conclusion:**

Compared with patients with liver metastasis alone, those with bone metastasis alone or both had poorer 3- and 5-year CSS. For patients with bone metastasis, undergoing chemotherapy was associated with better 3-year CSS. For patients with liver metastasis, receiving radiation was associated with worse 3-year CSS.

**Supplementary Information:**

The online version contains supplementary material available at 10.1186/s12887-024-04570-z.

## Background

Neuroblastoma is one of the most common malignant solid tumors in children that starts from the neural crest [1], accounting for around 15% of pediatric cancer-related deaths [2, 3]. Approximately 70% of patients with neuroblastoma exhibit metastasis [4, 5], more than half can experience distant metastasis at diagnosis [6]. Although the overall prognosis of neuroblastoma patients is good, patients with metastasis usually have poor survival even after radical treatment [7, 8]. The 5-year survival of high-risk children is lower than 50% [9].

Metastasis acts as an independent risk factor for survival in neuroblastoma, and bone and liver are the most common single metastatic sites of this disease [10]. Evidence demonstrated that two-thirds of neuroblastoma patients with bone metastasis had a primary site in the adrenal gland, with a 5-year survival rate of 62.1%, and age and tumor size were important factors affecting patients’ survival [11]. In addition, liver metastasis accounts for greater than 20% of neuroblastoma metastasis [12], but there is a lack of research on the clinical characteristics and prognosis of neuroblastoma patients with liver metastasis. Further studies are needed to investigate the differences in clinical characteristics, prognoses, and prognostic factors among neuroblastoma patients with different metastatic sites. Besides, treatment methods for the primary site (such as surgery or radiotherapy) exerted significant influences on the survival of neuroblastoma patients [13, 14]. Chemotherapy was shown to improve survival in patients suffering from neuroblastoma [15]. However, no relevant studies have been performed to explore the impacts of different therapeutic methods on the survival of neuroblastoma patients with different metastatic sites.

This study intended to probe into differences in clinical characteristics and prognoses of neuroblastoma patients with different metastatic sites (bone metastasis alone, liver metastasis alone, and both bone and liver metastasis), and assess the impacts of treatment methods on their survival, based on the Surveillance, Epidemiology, and End Results (SEER) database.

## Methods

### Study design and population

In this retrospective cohort study, data on neuroblastoma were extracted from the SEER 2010–2019. The c program of the National Cancer Institute (NCI) provides information on cancer incidence and survival, patient demographics, primary tumor site, treatment, etc., which was collected from population-based cancer registries, covering about 48% of the U.S. population (https://seer.cancer.gov/about/overview.html). Patients were included if they had (1) neuroblastoma [International Classification of Diseases for Oncology, Third Edition (ICD-O-3): 9490 or 9500]; (2) bone or liver metastasis; and (3) complete follow-up information. Patients (1) aged over 20 years at diagnosis; (2) diagnosed by autopsy or death certificate or only clinically diagnosed; 3) with missing important co-variables were excluded from this study. Since the data used in the current study were de-identified and freely accessible, the approval of the Institutional Review Board of Zhongshan City People’s Hospital was waived. The need for written informed consent was waived by the Institutional Review Board of Zhongshan City People’s Hospital due to the retrospective nature of the study. All methods were performed in accordance with the relevant guidelines and regulations.

### Data collection

Cancer registries received and collected data on cancer patients. The outcomes were 3-year cancer-specific survival (CSS) and 5-year CSS. Data about the following variables were also collected: metastasis type, age (years), sex, race, tumor site, tumor size (cm), grade, surgery for the primary site (no, yes), surgery for other regional or distant sites (no, yes), chemotherapy (no/unknown, yes), radiation (no/unknown, yes), brain metastasis (no, yes), lung metastasis (no, yes), follow-up time, and vital status. Metastasis type included liver metastasis, bone metastasis, and both liver and bone metastasis. Since the proportion of 1-year-old patients was small, and the age information in the SEER database was provided in an integer form, 12 or 18 months could not be used as the basis for the age grouping. Hence, the median age was used as the basis, and age was classified as < 3 and ≥ 3 years. Race included Black, White and others. Tumor site included the soft tissue (C47.0–47.9, C49.0–49.9), adrenal gland (C37.9, C74.0–75.9), retroperitoneum (C48.0-C48.8), and others. Tumor size was divided into < 5 cm, ≥ 5 cm and unknown. Grade was classified into Grade I/II/III (differentiated), Grade IV (undifferentiated or anaplastic) and unknown. The radiotherapy status was divided into “not receiving radiotherapy or having unknown information on radiotherapy (no/unknown)” and “receiving radiotherapy (yes)”.

### Statistical analysis

Measurement data were examined by the Kolmogorov–Smirnov test for normality. The measurement data of normal distribution were described by mean (standard deviation) [Mean (SE)], and comparison between two groups was conducted via the independent sample t-test; non-normal measurement data were reported as median and interquartile range [M (Q_1_, Q_3_)], and inter-group comparison was conducted using Mann–Whitney U rank sum test. Counting data were shown as the number of cases and constituent ratio [*n* (%)], and the Chi-square test was applied for between-group comparison.

All variables were incorporated into the univariable COX model to identify the variables related to CSS. The association between metastatic neuroblastoma and CSS was evaluated using multivariable COX risk proportional models. Model I was adjusted for age, sex, and race; Model II was adjusted for age, sex, race, tumor site, tumor size, grade, surgery for the primary site, surgery for other regional or distant sites, chemotherapy, and radiation. Then multivariable COX risk proportional models were established to assess the association between metastasis types and CSS. Model I was adjusted for age, sex, and race; Model II was adjusted for age, sex, race, tumor site, tumor size, grade, surgery for the primary site, surgery for other regional or distant sites, chemotherapy, radiation, brain metastasis, and lung metastasis. Hazard ratios (HRs) and 95% confidence intervals (CIs) were estimated. Then multivariable COX risk proportional models were established to assess the association between metastasis types and CSS. Model I was adjusted for age, sex, and race; Model II was adjusted for age, sex, race, tumor site, tumor size, grade, surgery for the primary site, surgery for other regional or distant sites, chemotherapy, radiation, brain metastasis, and lung metastasis. Hazard ratios (HRs) and 95% confidence intervals (CIs) were estimated. Further analysis was conducted by comparing the differences in results before and after the exclusion of patients with lung or brain metastasis alone. The number of neuroblastoma patients with bone or liver metastasis is shown in Supplementary Table 1.

Python 3.9 (Python Software Foundation, Delaware, USA) was used for data cleaning and missing value handling. SAS 9.4 (SAS Institute Inc., Cary, NC, USA) was adopted for model statistical analysis. Kaplan–Meier survival curves were drawn with R 4.0.3 (Institute for Statistics and Mathematics, Vienna, Austria). Two-sided *P* < 0.05 was deemed as statistically significant.

## Results

### Participant characteristics

**Fig. 1:**
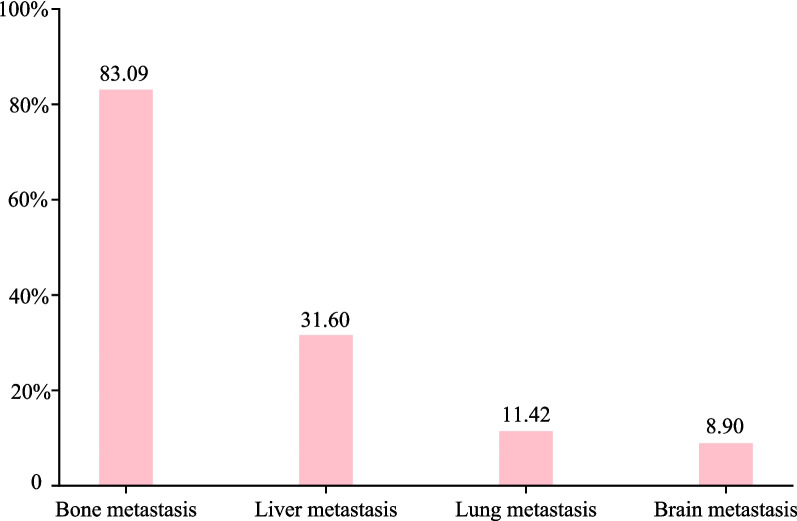
Histogram for proportions of metastatic types in neuroblastoma

**Fig. 2:**
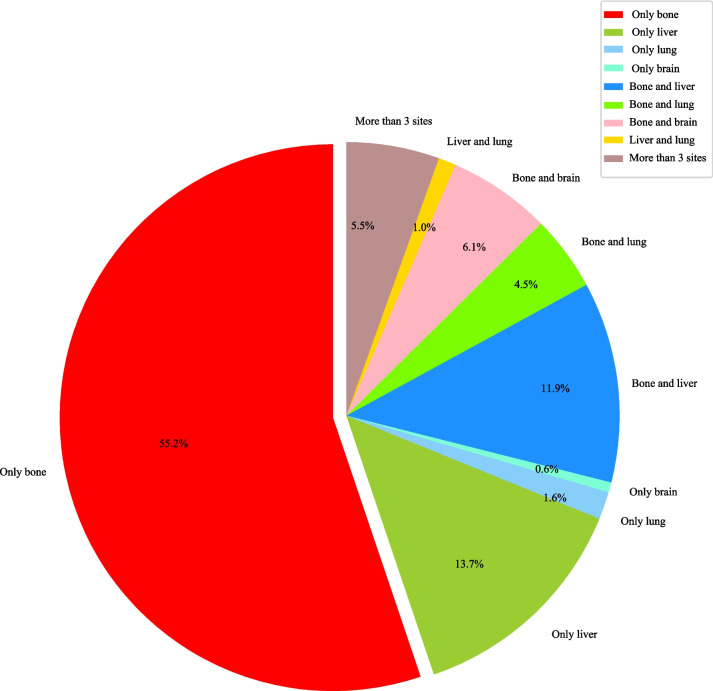
Pie chart for proportions of metastatic types in neuroblastoma

**Fig. 3:**
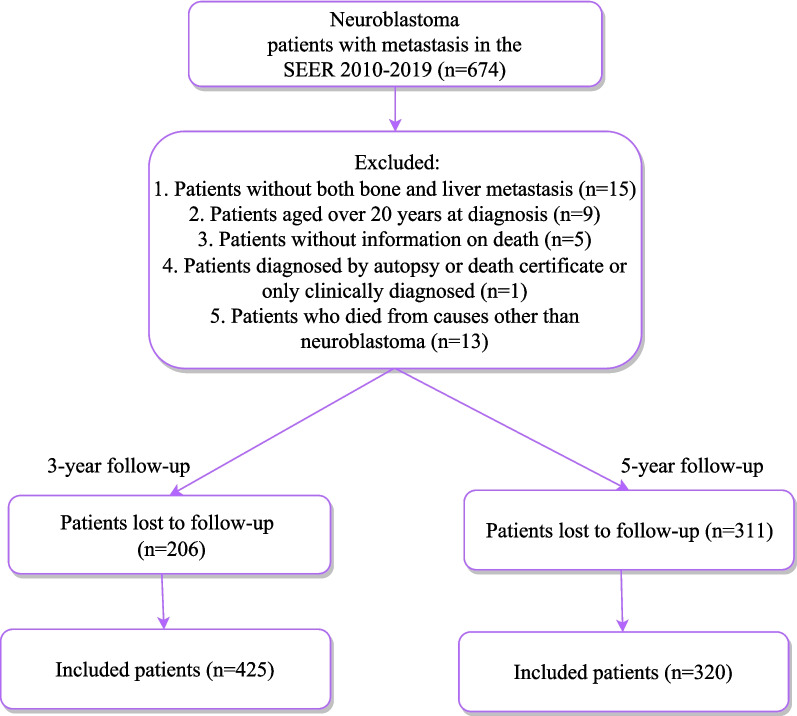
Flow chart of participant selection. SEER, the Surveillance, Epidemiology, and End Results

A total of 674 neuroblastoma patients with metastasis were identified from the SEER 2010–2019. According to Fig. 1, among the 674 patients, the proportions of bone metastasis and liver metastasis were high, both exceeding 30%; the proportions of lung metastasis and brain metastasis were relatively low. For patients with metastasis, the proportions of bone metastasis alone, liver metastasis alone, and both bone and liver metastasis were relatively high, accounting for 55.2%, 13.7%, and 11.9%, respectively (Fig. 2). Thus, this study focused on bone or liver metastasis. After excluding patients without both bone and liver metastasis (*n* = 15), aged over 20 years at diagnosis (*n* = 9), without information on death (*n* = 5), diagnosed by autopsy or death certificate or only clinically diagnosed (*n* = 1), and who died from causes other than neuroblastoma (*n* = 13, which was excluded due to the small sample size), 631 patients were included. Subsequently, patients lost to follow-up were ruled out. In the end, 425 were eligible for 3-year CSS analysis and 320 for 5-year CSS analysis. The flow chart of participant selection is shown in Fig. 3. The median follow-up time was 60.00 (19.00, 60.00) months. For 3-year follow-up, 306 (72%) patients were alive, and 119 (28%) died from neuroblastoma. Most patients had bone metastasis alone (68.00%), the tumor in the adrenal gland (69.65%), and a tumor size of ≥ 5 cm (51.06%). White people accounted for the majority (73.88%). Significant differences were found in metastasis type, tumor size, surgery for other regional or distant sites, brain metastasis, lung metastasis, and follow-up time between the alive and dead groups (all *P* < 0.05). For 5-year follow-up, 180 (56.25%) patients were alive, and 140 (43.75%) died from neuroblastoma. The general characteristics of patients with 5-year follow-up at maximum were similar to those of patients with maximum 3-year follow-up. Table 1 exhibits the features of the included neuroblastoma patients.

**Table 1:** Characteristics of the included neuroblastoma patients

Variables	3-year follow-up					5-year follow-up				
	Total (*n* = 425)	Alive (*n* = 306)	Dead (*n* = 119)	Statistics	*P*	Total (*n* = 320)	Alive (*n* = 180)	Dead (*n* = 140)	Statistics	*P*
Metastasis type, *n* (%)				χ^2^ = 7.89	0.019				χ^2^ = 8.98	0.011
Liver	62 (14.59)	52 (16.99)	10 (8.40)			44 (13.75)	33 (18.33)	11 (7.86)		
Bone	289 (68.00)	208 (67.97)	81 (68.07)			223 (69.69)	123 (68.33)	100 (71.43)		
Liver and Bone	74 (17.41)	46 (15.03)	28 (23.53)			53 (16.56)	24 (13.33)	29 (20.71)		
Age, *n* (%)				χ^2^ = 0.01	0.923				χ^2^ = 0.24	0.626
< 3 years	252 (59.29)	181 (59.15)	71 (59.66)			190 (59.38)	109 (60.56)	81 (57.86)		
≥ 3 years	173 (40.71)	125 (40.85)	48 (40.34)			130 (40.63)	71 (39.44)	59 (42.14)		
Sex, *n* (%)				χ^2^ = 0.24	0.627				χ^2^ = 0.06	0.810
Female	192 (45.18)	136 (44.44)	56 (47.06)			151 (47.19)	86 (47.78)	65 (46.43)		
Male	233 (54.82)	170 (55.56)	63 (52.94)			169 (52.81)	94 (52.22)	75 (53.57)		
Race, *n* (%)				χ^2^ = 0.09	0.957				χ^2^ = 0.55	0.759
Black	64 (15.06)	46 (15.03)	18 (15.13)			56 (17.50)	34 (18.89)	22 (15.71)		
White	314 (73.88)	227 (74.18)	87 (73.11)			228 (71.25)	126 (70.00)	102 (72.86)		
Others	47 (11.06)	33 (10.78)	14 (11.76)			36 (11.25)	20 (11.11)	16 (11.43)		
Tumor site, *n* (%)				χ^2^ = 5.14	0.162				χ^2^ = 5.72	0.126
Soft tissue	68 (16.00)	56 (18.30)	12 (10.08)			51 (15.94)	36 (20.00)	15 (10.71)		
Adrenal gland	296 (69.65)	210 (68.63)	86 (72.27)			226 (70.63)	123 (68.33)	103 (73.57)		
Retroperitoneum	39 (9.18)	25 (8.17)	14 (11.76)			28 (8.75)	13 (7.22)	15 (10.71)		
Others	22 (5.18)	15 (4.90)	7 (5.88)			15 (4.69)	8 (4.44)	7 (5.00)		
Tumor size, *n* (%)				χ^2^ = 10.05	0.007				χ^2^ = 15.74	< 0.001
< 5 cm	71 (16.71)	62 (20.26)	9 (7.56)			49 (15.31)	38 (21.11)	11 (7.86)		
≥ 5 cm	217 (51.06)	151 (49.35)	66 (55.46)			184 (57.50)	105 (58.33)	79 (56.43)		
Unknown	137 (32.24)	93 (30.39)	44 (36.97)			87 (27.19)	37 (20.56)	50 (35.71)		
Grade, n (%)				χ^2^ = 4.96	0.084				χ^2^ = 4.85	0.089
Grade I/II/III	223 (52.47)	168 (54.90)	55 (46.22)			167 (52.19)	101 (56.11)	66 (47.14)		
Grade IV	30 (7.06)	17 (5.56)	13 (10.92)			24 (7.50)	9 (5.00)	15 (10.71)		
Unknown	172 (40.47)	121 (39.54)	51 (42.86)			129 (40.31)	70 (38.89)	59 (42.14)		
Surgery for the primary site, *n* (%)				χ^2^ = 0.26	0.609				χ^2^ = 1.27	0.260
No	111 (26.12)	82 (26.80)	29 (24.37)			76 (23.75)	47 (26.11)	29 (20.71)		
Yes	314 (73.88)	224 (73.20)	90 (75.63)			244 (76.25)	133 (73.89)	111 (79.29)		
Surgery for other regional or distant sites, *n* (%)				χ^2^ = 4.19	0.041				χ^2^ = 4.49	0.034
No	338 (79.53)	251 (82.03)	87 (73.11)			255 (79.69)	151 (83.89)	104 (74.29)		
Yes	87 (20.47)	55 (17.97)	32 (26.89)			65 (20.31)	29 (16.11)	36 (25.71)		
Chemotherapy, n (%)				-	0.510				-	0.761
No/unknown	11 (2.59)	7 (2.29)	4 (3.36)			11 (3.44)	7 (3.89)	4 (2.86)		
Yes	414 (97.41)	299 (97.71)	115 (96.64)			309 (96.56)	173 (96.11)	136 (97.14)		
Radiation, *n* (%)				χ^2^ = 0.01	0.945				χ^2^ = 0.22	0.642
No/unknown	231 (54.35)	166 (54.25)	65 (54.62)			167 (52.19)	96 (53.33)	71 (50.71)		
Yes	194 (45.65)	140 (45.75)	54 (45.38)			153 (47.81)	84 (46.67)	69 (49.29)		
Brain metastasis, *n* (%)				χ^2^ = 5.69	0.017				χ^2^ = 6.79	0.009
No	384 (90.35)	283 (92.48)	101 (84.87)			286 (89.38)	168 (93.33)	118 (84.29)		
Yes	41 (9.65)	23 (7.52)	18 (15.13)			34 (10.63)	12 (6.67)	22 (15.71)		
Lung metastasis, n (%)				χ^2^ = 10.65	0.001				χ^2^ = 4.18	0.041
No	377 (88.71)	281 (91.83)	96 (80.67)			279 (87.19)	163 (90.56)	116 (82.86)		
Yes	48 (11.29)	25 (8.17)	23 (19.33)			41 (12.81)	17 (9.44)	24 (17.14)		
Follow-up time, months, M (Q_1_, Q_3_)	36.00 (27.00, 36.00)	36.00 (36.00, 36.00)	14.00 (10.00, 22.00)	Z = -20.23	< 0.001	60.00 (19.00, 60.00)	60.00 (60.00, 60.00)	17.00 (10.00, 27.00)	Z = -16.93	< 0.001

-: Fisher’s precision probability test

*M* Median, *Q*_*1*_ 1st Quartile, *Q*_*3*_ 3rd Quartile

### Characteristics of patients with bone or liver metastasis

After 3-year follow-up, 566 (57.11%) patients had non-metastatic neuroblastoma, and 425 (42.89%) had bone or liver metastasis. Compared with patients with non-metastatic neuroblastoma, those with metastatic neuroblastoma tended to be ≥ 3 years old (40.71% vs 31.27%), have the tumor in the adrenal gland (69.65% vs 36.40%) and a tumor size of ≥ 5 cm (51.06% vs 44.17%), undergo surgery for other regional or distant sites (20.47% vs 5.32%), chemotherapy (97.41% vs 52.30%) and radiation (45.65% vs 13.96%), and die (28.00% vs 9.19%) (all *P* < 0.05). After 5-year follow-up, 370 (53.62%) patients had non-metastatic neuroblastoma, and 320 (46.38%) had bone or liver metastasis. Significant differences were also observed in tumor site, tumor size, surgery for the primary site, surgery for other regional or distant sites, chemotherapy, radiation, and vital status between patients with non-metastatic and metastatic neuroblastoma (all *P* < 0.05) (Supplementary Table 2). As illustrated in Table 2, for 3-year follow-up, 62 (14.59%) patients had liver metastasis alone, 289 (68%) had bone metastasis alone, and 74 (17.41%) had both liver and bone metastasis. There were significant differences in age, tumor site, tumor size, surgery for the primary site, chemotherapy, radiation, brain metastasis, lung metastasis, and vital status among patients with liver metastasis alone, bone metastasis alone, and both liver and bone metastasis (all *P* < 0.05). The 3-year CSS rate of patients with liver metastasis alone, bone metastasis alone, and both liver and bone metastasis was 83.87%, 71.97%, and 62.16%, respectively. For 5-year follow-up, 44 (13.75%) patients had liver metastasis alone, 223 (69.69%) had bone metastasis alone, and 53 (16.56%) had both liver and bone metastasis. Significant differences were observed in age, tumor size, surgery for the primary site, chemotherapy, radiation, brain metastasis, lung metastasis, and vital status among patients with liver metastasis alone, bone metastasis alone, and both liver and bone metastasis (all *P* < 0.05). The 5-year CSS rate of patients with liver metastasis alone, bone metastasis alone, and both liver and bone metastasis was 75.00%, 55.16%, and 45.28%, respectively.

**Table 2:** Characteristics of neuroblastoma patients with bone or liver metastasis

Variables	3-year follow-up					5-year follow-up				
	Liver metastasis alone (*n* = 62)	Bone metastasis alone (*n* = 289)	Bone metastasis and liver metastasis (*n* = 74)	Statistics	*P*	Liver metastasis alone(*n* = 44)	Bone metastasis alone (*n* = 223)	Bone metastasis and liver metastasis (*n* = 53)	Statistics	*P*
Age, *n* (%)				χ^2^ = 46.59	< 0.001				χ^2^ = 29.56	< 0.001
< 3 years old	60 (96.77)	145 (50.17)	47 (63.51)			42 (95.45)	115 (51.57)	33 (62.26)		
≥ 3 years old	2 (3.23)	144 (49.83)	27 (36.49)			2 (4.55)	108 (48.43)	20 (37.74)		
Sex, *n* (%)				χ^2^ = 0.57	0.752				χ^2^ = 1.08	0.583
Female	30 (48.39)	127 (43.94)	35 (47.30)			23 (52.27)	101 (45.29)	27 (50.94)		
Male	32 (51.61)	162 (56.06)	39 (52.70)			21 (47.73)	122 (54.71)	26 (49.06)		
Race, *n* (%)				χ^2^ = 5.29	0.259				χ^2^ = 4.10	0.393
Black	4 (6.45)	49 (16.96)	11 (14.86)			4 (9.09)	44 (19.73)	8 (15.09)		
White	52 (83.87)	206 (71.28)	56 (75.68)			36 (81.82)	153 (68.61)	39 (73.58)		
Others	6 (9.68)	34 (11.76)	7 (9.46)			4 (9.09)	26 (11.66)	6 (11.32)		
Tumor site, *n* (%)				χ^2^ = 14.95	0.021				-	0.149
Soft tissue	7 (11.29)	52 (17.99)	9 (12.16)			4 (9.09)	38 (17.04)	9 (16.98)		
Adrenal gland	51 (82.26)	190 (65.74)	55 (74.32)			36 (81.82)	153 (68.61)	37 (69.81)		
Retroperitoneum	1 (1.61)	29 (10.03)	9 (12.16)			1 (2.27)	20 (8.97)	7 (13.21)		
Others	3 (4.84)	18 (6.23)	1 (1.35)			3 (6.82)	12 (5.38)	0 (0.00)		
Tumor size, *n* (%)				χ^2^ = 18.30	0.001				χ^2^ = 13.79	0.008
< 5 cm	20 (32.26)	41 (14.19)	10 (13.51)			14 (31.82)	31 (13.90)	4 (7.55)		
≥ 5 cm	20 (32.26)	162 (56.06)	35 (47.30)			18 (40.91)	135 (60.54)	31 (58.49)		
Unknown	22 (35.48)	86 (29.76)	29 (39.19)			12 (27.27)	57 (25.56)	18 (33.96)		
Grade, *n* (%)				χ^2^ = 2.27	0.687				χ^2^ = 5.18	0.270
Grade I/II/III	33 (53.23)	156 (53.98)	34 (45.95)			24 (54.55)	122 (54.71)	21 (39.62)		
Grade IV	3 (4.84)	20 (6.92)	7 (9.46)			2 (4.55)	18 (8.07)	4 (7.55)		
Unknown	26 (41.94)	113 (39.10)	33 (44.59)			18 (40.91)	83 (37.22)	28 (52.83)		
Surgery for the primary site, *n* (%)				χ^2^ = 31.41	< 0.001				χ^2^ = 18.30	< 0.001
No	32 (51.61)	54 (18.69)	25 (33.78)			20 (45.45)	39 (17.49)	17 (32.08)		
Yes	30 (48.39)	235 (81.31)	49 (66.22)			24 (54.55)	184 (82.51)	36 (67.92)		
Surgery for other regional or distant sites, *n* (%)				χ^2^ = 0.90	0.639				χ^2^ = 0.73	0.695
No	49 (79.03)	233 (80.62)	56 (75.68)			35 (79.55)	180 (80.72)	40 (75.47)		
Yes	13 (20.97)	56 (19.38)	18 (24.32)			9 (20.45)	43 (19.28)	13 (24.53)		
Chemotherapy, *n* (%)				-	< 0.001				-	< 0.001
No/unknown	7 (11.29)	3 (1.04)	1 (1.35)			7 (15.91)	3 (1.35)	1 (1.89)		
Yes	55 (88.71)	286 (98.96)	73 (98.65)			37 (84.09)	220 (98.65)	52 (98.11)		
Radiation, *n* (%)				χ^2^ = 44.24	< 0.001				χ^2^ = 29.91	< 0.001
No/unknown	57 (91.94)	132 (45.67)	42 (56.76)			39 (88.64)	98 (43.95)	30 (56.60)		
Yes	5 (8.06)	157 (54.33)	32 (43.24)			5 (11.36)	125 (56.05)	23 (43.40)		
Brain metastasis, *n* (%)				χ^2^ = 8.28	0.016				χ^2^ = 6.12	0.047
No	62 (100.00)	258 (89.27)	64 (86.49)			44 (100.00)	196 (87.89)	46 (86.79)		
Yes	0 (0.00)	31 (10.73)	10 (13.51)			0 (0.00)	27 (12.11)	7 (13.21)		
Lung metastasis, *n* (%)				χ^2^ = 18.50	< 0.001				χ^2^ = 17.17	< 0.001
No	57 (91.94)	265 (91.70)	55 (74.32)			40 (90.91)	202 (90.58)	37 (69.81)		
Yes	5 (8.06)	24 (8.30)	19 (25.68)			4 (9.09)	21 (9.42)	16 (30.19)		
Vital status, *n* (%)				χ^2^ = 7.89	0.019				χ^2^ = 8.98	0.011
Alive	52 (83.87)	208 (71.97)	46 (62.16)			33 (75.00)	123 (55.16)	24 (45.28)		
Dead	10 (16.13)	81 (28.03)	28 (37.84)			11 (25.00)	100 (44.84)	29 (54.72)		

-: Fisher’s precision probability test

### Survival of patients with bone or liver metastasis

**Fig. 4:**
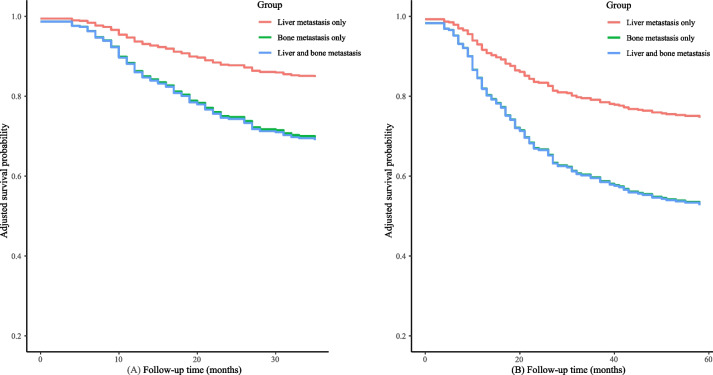
CSS of neuroblastoma patients with bone or liver metastasis. (**A**) Three-year CSS; **B** Five-year CSS. Age, sex, race, tumor site, tumor size, grade, surgery for the primary site, surgery for other regional or distant sites, chemotherapy, radiation, brain metastasis, and lung metastasis were adjusted for CSS, cancer-specific survival

According to Supplementary Table 3, patients with metastatic neuroblastoma had a significantly worse 3- and 5-year CSS than those with non-metastatic neuroblastoma after controlling for age, sex, race, tumor site, tumor size, grade, surgery for the primary site, surgery for other regional or distant sites, chemotherapy, and radiation. After adjusting for age, sex, race, tumor site, tumor size, grade, surgery for the primary site, surgery for other regional or distant sites, chemotherapy, radiation, brain metastasis, and lung metastasis, compared with neuroblastoma patients who had liver metastasis alone, patients who have bone metastasis alone (HR = 2.30, 95%CI: 1.10–4.82, *P* = 0.028) or both (HR = 2.35, 95%CI: 1.06–5.20, *P* = 0.035) had significantly poorer 3-year CSS; patients with bone metastasis alone (HR = 2.32, 95%CI: 1.14–4.70, *P* = 0.020) or both liver and bone metastasis (HR = 2.33, 95%CI: 1.07–5.07, *P* = 0.032) exhibited significantly worse 5-year CSS than those with liver metastasis alone (Table 3). Figure 4 demonstrated no significant differences in 3-year and 5-year CSS between patients with bone metastasis alone and with both liver and bone metastasis, indicating relatively poorer CSS in bone metastasis.

**Table 3:** CSS of neuroblastoma patients with bone or liver metastasis

Variables	Model I		Model II	
	HR (95%CI)	*P*	HR (95%CI)	*P*
3-year CSS				
Metastasis type				
Liver	Ref		Ref	
Bone	1.99 (1.01–3.94)	0.049	2.30 (1.10–4.82)	0.028
Liver and bone	2.82 (1.36–5.86)	0.006	2.35 (1.06–5.20)	0.035
5-year CSS				
Metastasis type				
Liver	Ref		Ref	
Bone	2.11 (1.10–4.03)	0.024	2.32 (1.14–4.70)	0.020
Liver and bone	2.90 (1.43–5.86)	0.003	2.33 (1.07–5.07)	0.032

Model I was adjusted for age, sex, and race

Model II was adjusted for age, sex, race, tumor site, tumor size, grade, surgery for the primary site, surgery for other regional or distant sites, chemotherapy, radiation, brain metastasis, and lung metastasis

*CSS* cancer-specific survival, *Ref* reference, *HR* hazard ratio, *CI* confidence interval

In patients without brain metastasis, after adjusting for age, sex, race, tumor site, tumor size, grade, surgery for the primary site, surgery for other regional or distant sites, chemotherapy, radiation, and lung metastasis, bone metastasis alone (HR = 2.42, 95%CI: 1.15–5.09, *P* = 0.020) or both liver and bone metastasis (HR = 2.52, 95%CI: 1.12–5.64, *P* = 0.025) were associated with significantly worse 3-year CSS than liver metastasis alone; patients with bone metastasis alone (HR = 2.35, 95%CI: 1.15–4.80, *P* = 0.019) or both liver and bone metastasis (HR = 2.27, 95%CI: 1.04–4.99, *P* = 0.041) had significantly poorer 5-year CSS than those with liver metastasis alone. Among patients without lung metastasis, after controlling for age, sex, race, tumor site, tumor size, grade, surgery for the primary site, surgery for other regional or distant sites, chemotherapy, radiation, and brain metastasis, patients with both liver and bone metastasis had significantly poorer 5-year CSS than those with liver metastasis alone (HR = 2.56, 95%CI: 1.11–5.87, *P* = 0.027) (Table 4).

**Table 4:** CSS of neuroblastoma patients after excluding patients with brain or lung metastasis alone

Excluding patients with brain metastasis alone	3-year CSS			5-year CSS		
	Sample size	HR (95%CI)	*P*	Sample size	HR (95%CI)	*P*
Study population	384			286		
Metastasis type						
Liver	62	Ref		44	Ref	
Bone	258	2.42 (1.15–5.09)	0.020	196	2.35 (1.15–4.80)	0.019
Liver and bone	64	2.52 (1.12–5.64)	0.025	46	2.27 (1.04–4.99)	0.041
Excluding patients with lung metastasis alone	Sample size	HR (95%CI)	*P*	Sample size	HR (95%CI)	*P*
Study population	377			279		
Metastasis type						
Liver	57	Ref		40	Ref	
Bone	265	1.64 (0.73–3.66)	0.232	202	1.77 (0.83–3.78)	0.137
Liver and bone	55	2.27 (0.96–5.40)	0.063	37	2.56 (1.11–5.87)	0.027

When excluding patients with brain metastasis alone, age, sex, race, tumor site, tumor size, grade, surgery for the primary site, surgery for other regional or distant sites, chemotherapy, radiation, and lung metastasis were adjusted for

When excluding patients with lung metastasis alone, age, sex, race, tumor site, tumor size, grade, surgery for the primary site, surgery for other regional or distant sites, chemotherapy, radiation, and brain metastasis were adjusted for

*CSS* cancer-specific survival, *Ref* reference, *HR* hazard ratio, *CI* confidence interval

### Association between treatments and CSS in patients with bone or liver metastasis

In patients with bone metastasis, those receiving chemotherapy had significantly better 3-year CSS than those without chemotherapy, after adjusting for age, sex, race, tumor size, tumor site, grade, brain metastasis, lung metastasis, and liver metastasis (HR = 0.24, 95%CI: 0.07–0.75, *P* = 0.014). Among patients with liver metastasis, after controlling for age, sex, race, tumor size, tumor site, grade, brain metastasis, lung metastasis, and bone metastasis, receiving radiation was associated with significantly worse 3-year CSS (HR = 2.00, 95%CI: 1.05–3.81, *P* = 0.035). No significant association was found between surgery for the primary site and surgery for other regional or distant sites and CSS in patients with bone metastasis or liver metastasis (all *P* > 0.05) (Table 5).

**Table 5:** Association between treatments and CSS in neuroblastoma patients with bone or liver metastasis

Variables	Bone metastasis				Liver metastasis			
	3-year CSS (*n* = 363)		5-year CSS (*n* = 276)		3-year CSS (*n* = 136)		5-year CSS (*n* = 97)	
	HR (95%CI)	*P*	HR (95%CI)	*P*	HR (95%CI)	*P*	HR (95%CI)	*P*
Surgery for the primary site								
No	Ref		Ref		Ref		Ref	
Yes	0.89 (0.57–1.39)	0.613	0.97 (0.63–1.50)	0.893	1.23 (0.64–2.38)	0.537	1.10 (0.58–2.11)	0.770
Surgery for other regional or distant sites								
No	Ref		Ref		Ref		Ref	
Yes	1.48 (0.97–2.27)	0.070	1.43 (0.96–2.13)	0.080	1.36 (0.68–2.75)	0.388	1.46 (0.74–2.87)	0.277
Chemotherapy								
No/unknown	Ref		Ref		Ref		Ref	
Yes	0.24 (0.07–0.75)	0.014	0.37 (0.12–1.16)	0.089	0.99 (0.24–4.13)	0.993	1.65 (0.40–6.86)	0.489
Radiation								
No/unknown	Ref		Ref		Ref		Ref	
Yes	0.76 (0.52–1.11)	0.155	0.80 (0.57–1.13)	0.213	2.00 (1.05–3.81)	0.035	1.72 (0.91–3.24)	0.095

For bone metastasis: age, sex, race, tumor size, tumor site, grade, brain metastasis, lung metastasis, and liver metastasis were adjusted for;

For liver metastasis: age, sex, race, tumor size, tumor site, grade, brain metastasis, lung metastasis, and bone metastasis were adjusted for

*CSS*, cancer-specific survival, *Ref* reference, *HR* hazard ratio, *CI* confidence interval

## Discussion

To the best of our knowledge, this study was the first to evaluate differences in clinical characteristics and prognoses among neuroblastoma patients with bone metastasis alone, liver metastasis alone, and both bone and liver metastasis, and the influences of different therapeutic methods on survival. The results demonstrated significant differences in age, tumor size, surgery for the primary site, chemotherapy, radiation, brain metastasis, lung metastasis, and vital status between patients with liver metastasis alone, bone metastasis alone, and both liver and bone metastasis. In contrast to patients with liver metastasis alone, patients with bone metastasis alone or both had significantly poorer 3- and 5-year CSS. Besides, for patients with bone metastasis, those receiving chemotherapy had significantly better 3-year CSS than those without chemotherapy. For patients with liver metastasis, receiving radiation was associated with significantly worse 3-year CSS. These findings may assist in understanding the disease features (including survival) of metastatic neuroblastoma and the role of treatments in patients with metastasis, which might further facilitate timely interventions to get favorable prognoses.

He et al. [11] investigated the clinical characteristics, survival and prognostic factors of neuroblastoma patients with bone metastasis. Another study by Liu et al. [10] explored the metastasis pattern of neuroblastoma, overall survival and CSS of neuroblastoma patients with different metastatic sites, and risk factors for metastasis. The current study focused on neuroblastoma patients with bone or liver metastasis, since bone and liver metastasis accounted for relatively high proportions, and assessed clinical features, 3- and 5-year CSS, and therapeutic approaches among patients with bone and liver metastasis. Patients with bone metastasis alone and both liver and bone metastasis tended to be older than ≥ 3 years, have a tumor size of ≥ 5 cm, receive surgery for the primary site, chemotherapy, and radiation, and have brain metastasis, compared with those with liver metastasis alone. Clinicians could pay attention to these characteristics, and provide early counseling and management measures for people at risk of metastasizing to different sites. It was demonstrated that age over 1 year and tumors of 5–10 cm were correlated with an increased risk of bone metastasis [10]. A previous study proposed that age, tumor biology and survival were associated with the metastasis pattern of neuroblastoma. The biological characteristics of the tumor change with age, leading to differences in the metastasis ability or tumor affinity with specific sites [16]. Monte et al. [17] also revealed that chemotherapy and/or radiotherapy changed the metastasis pattern of neuroblastic tumors. A previous study reported that neuroblastoma patients with bone metastasis had a CSS of 64.1% [18]. We found more information on CSS of patients with different metastatic sites: the 3-year CSS rate of patients with liver metastasis alone, bone metastasis alone, and both liver and bone metastasis was 83.87%, 71.97%, and 62.16%, separately; the 5-year CSS rate of patients with liver metastasis alone, bone metastasis alone, and both liver and bone metastasis was 75.00%, 55.16%, and 45.28%, respectively. Evidence from larger sample sizes is necessitated for CSS rate corroboration. Further, compared with liver metastasis alone, bone metastasis alone or both were associated with significantly worse 3- and 5-year CSS, and patients with bone metastasis (combined with liver metastasis or not) exhibited poorer CSS, according to this study. A possible explanation for the better 3- and 5-year CSS in the liver metastasis group is the presence of more 4S stage neuroblastoma in the liver metastasis group. In patients without brain metastasis, the association between bone or liver metastasis and 3- and 5-year CSS was consistent with the above, while for patients without lung metastasis, merely both liver and bone metastasis was associated with significantly worse 5-year CSS than liver metastasis alone. This may be attributed to the relatively small sample size for our analysis. Future large-scale investigations should be conducted to validate these findings. Liu et al. [10] illustrated similar CSS in the bone metastasis alone group, liver metastasis alone group, and the both bone and liver metastasis group, but covariables were not taken into consideration in their research. We have controlled for potential confounding factors in this analysis to minimize their effect.

As regards the role of different treatments in survival of neuroblastoma patients, this study showed that for patients with bone metastasis, those with chemotherapy had better 3-year CSS than those without, and for patients with liver metastasis, receiving radiation was associated with worse 3-year CSS, suggesting that chemotherapy may confer survival benefit in neuroblastoma patients with bone metastasis. Induction chemotherapy (IC) can shrink the tumor, and lower the risk of further metastasis in neuroblastoma [18, 19]. Increased dose intensity in chemotherapy was related to greater response and survival of neuroblastoma patients [20]. More research is required to verify the protective effect of chemotherapy on survival in metastatic neuroblastoma. Concerning the unfavorable impact of radiotherapy in patients with liver metastasis, radiation treatment may bring late side effects for individuals with neuroblastoma, such as hypothyroidism, lung and heart abnormalities, musculoskeletal abnormalities, and growth and developmental failure [21, 22]. Another possible explanation is that radiation can elevate the risk of secondary neoplasms, because vesicles secreted from irradiated neuroblastoma cells promote proliferation and invasion related to the epithelial-to-mesenchymal transition in non-irradiated cells [23], which may be associated with worse survival in 3 years. Besides, complications after radiotherapy included hypertension, veno-occlusive disease, nerve lesion, and bowel obstructions [21]. Patients with neuroblastoma undergoing radiation may be at a higher risk of vascular injury from the tumor and surgery, since they were chosen to receive radiotherapy for the more invasive, surgically challenging tumors [24], posing a threat to survival. A prior review showed that palliative radiation contributed to high response rates and symptomatic relief, whereas survival is unsatisfactory in metastatic neuroblastoma [25]. Notably, the sample size used to analyze the role of radiotherapy in patients with liver metastasis is relatively small in this study. At present, the relationship between radiotherapy and CSS among neuroblastoma patients with bone or liver metastasis is under-researched, which necessitates large-scale studies in the future. As to surgery for neuroblastoma, it remains an important component in treating high-risk neuroblastoma and controlling the localized tumor [14]. Kubota [26] put forward that the influence of surgery varies by different clinical situations, and the benefits of surgery to survival in high-risk neuroblastoma may be limited. Neuroblastoma with macroscopic residual tumor died within 18 months after surgery due to systemic metastasis [27], and surgical eradication may be crucial [28]. We found no significant association between surgery for the primary site and surgery for other regional or distant sites and CSS in neuroblastoma patients with bone metastasis or liver metastasis. A small sample size may be an explanation. For another, survival advantages brought by surgery may be offset by adverse effects from a great incidence of surgical complications and the level of resection.

Using this nationally representative data, differences in clinical features and CSS and impacts of treatments on CSS among neuroblastoma patients with bone or liver metastasis were exhibited. Close attention should be paid to patients with bone metastasis, and early interventions should be taken when necessary. Adjustment of therapeutic methods such as radiotherapy may improve outcomes in neuroblastoma patients with bone or liver metastasis. Some limitations should be noted. First, this study had a retrospective study, which may introduce selection bias. Second, the treatment protocol of NB mainly included chemotherapy, radiotherapy, and surgery, and more detailed treatments were not available from the SEER database. Besides, since the SEER database did not provide all the information required for neuroblastoma risk stratification by Children’s Oncology Group (COG) and SIOP, such as International Neuroblastoma Staging System (INSS) stage, MYCN status and DNA ploidy, the risk of patients with neuroblastoma could not be determined in this study. Third, information on different metastatic sites was collected only after 2010 in the SEER, and the data used in this study came from the SEER 2010–2019, which may result in insufficient follow-up time. Ultimately, the findings of this study may not be generalizable to populations in other countries.

## Conclusion

Compared with liver metastasis alone, bone metastasis alone or both was associated with poorer 3- and 5-year CSS. For patients with bone metastasis, those with chemotherapy had better 3-year CSS than those without. For patients with liver metastasis, receiving radiation was associated with worse 3-year CSS. More studies are warranted to support these findings.

### Supplementary Information

**Additional file 1:** **Supplementary Table 1.** Sample size of neuroblastoma patients with bone or liver metastasis.

**Additional file 2:** **Supplementary Table 2.** Characteristics of patients with metastatic neuroblastoma and non-metastatic neuroblastoma.

**Additional file 3:** **Supplementary Table 3.** CSS of patients with metastatic neuroblastoma and non-metastatic neuroblastoma.

## Data Availability

The datasets generated and/or analyzed during the current study are available in the SEER database, https://seer.cancer.gov/.

## Abbreviations

SEER: Surveillance, Epidemiology, and End Results
NCI: National Cancer Institute
ICD-O-3: International Classification of Diseases for Oncology, Third Edition
CSS: Cancer-specific survival
Mean (SE): Mean (standard deviation)
M (Q_1_, Q_3_): Median and interquartile range
HRs: Hazard ratios
CIs: Confidence intervals
