# Supplementary material for: Clinical characteristics and prognoses in pediatric neuroblastoma with bone or liver metastasis: data from the SEER 2010–2019
Source: BMC Pediatr. 2024 Mar 7;24:162. doi: 10.1186/s12887-024-04570-z (PMC10921780; doi:10.1186/s12887-024-04570-z)
Supplement: Supplementary file 1 — Additional file 1: Supplementary Table 1. Sample size of neuroblastoma patients with bone or liver metastasis. [file 12887_2024_4570_MOESM1_ESM.docx]

Supplementary Table 1 Sample size of neuroblastoma patients with bone or liver metastasis.

| Patients | 3-year CSS | 5-year CSS |
| --- | --- | --- |
| Without brain metastasis and without lung metastasis | 342 (80%) | 250 (78%) |
| With brain metastasis and without lung metastasis | 35 (8%) | 29 (9%) |
| Without brain metastasis and with lung metastasis | 42 (10%) | 36 (11%) |
| With brain metastasis and with lung metastasis | 6 (2%) | 5 (2%) |
| Total | 425 | 320 |

CSS, cancer-specific survival.
