# Supplementary material for: Clinical characteristics and prognoses in pediatric neuroblastoma with bone or liver metastasis: data from the SEER 2010–2019
Source: BMC Pediatr. 2024 Mar 7;24:162. doi: 10.1186/s12887-024-04570-z (PMC10921780; doi:10.1186/s12887-024-04570-z)
Supplement: Supplementary file 2 — Additional file 2: Supplementary Table 2. Characteristics of patients with metastatic neuroblastoma and non-metastatic neuroblastoma. [file 12887_2024_4570_MOESM2_ESM.docx]

Supplementary Table 2 Characteristics of patients with metastatic neuroblastoma and non-metastatic neuroblastoma.

| Variables | 3-year follow-up | | | | | 5-year follow-up | | | | |
| --- | --- | --- | --- | --- | --- | --- | --- | --- | --- | --- |
|  | Total (n=991) | Non-metastasis (n=566) | Bone or liver metastasis (n=425) | Statistics | *P* | Total (n=690) | Non-metastasis (n=370) | Bone or liver metastasis (n=320) | Statistics | *P* |
| Age, n (%) |  |  |  | χ^2^=9.46 | 0.002 |  |  |  | χ^2^=3.73 | 0.053 |
| < 3 years old | 641 (64.68) | 389 (68.73) | 252 (59.29) |  |  | 436 (63.19) | 246 (66.49) | 190 (59.38) |  |  |
| ≥ 3 years old | 350 (35.32) | 177 (31.27) | 173 (40.71) |  |  | 254 (36.81) | 124 (33.51) | 130 (40.63) |  |  |
| Sex, n (%) |  |  |  | χ^2^=2.97 | 0.085 |  |  |  | χ^2^=0.06 | 0.809 |
| Female | 479 (48.34) | 287 (50.71) | 192 (45.18) |  |  | 329 (47.68) | 178 (48.11) | 151 (47.19) |  |  |
| Male | 512 (51.66) | 279 (49.29) | 233 (54.82) |  |  | 361 (52.32) | 192 (51.89) | 169 (52.81) |  |  |
| Race, n (%) |  |  |  | χ^2^=0.61 | 0.738 |  |  |  | χ^2^=1.61 | 0.447 |
| Black | 140 (14.13) | 76 (13.43) | 64 (15.06) |  |  | 108 (15.65) | 52 (14.05) | 56 (17.50) |  |  |
| White | 737 (74.37) | 423 (74.73) | 314 (73.88) |  |  | 505 (73.19) | 277 (74.86) | 228 (71.25) |  |  |
| Others | 114 (11.50) | 67 (11.84) | 47 (11.06) |  |  | 77 (11.16) | 41 (11.08) | 36 (11.25) |  |  |
| Tumor site, n (%) |  |  |  | χ^2^=114.94 | <0.001 |  |  |  | χ^2^=93.18 | <0.001 |
| Adrenal gland | 502 (50.66) | 206 (36.40) | 296 (69.65) |  |  | 358 (51.88) | 132 (35.68) | 226 (70.63) |  |  |
| Soft tissue | 269 (27.14) | 201 (35.51) | 68 (16.00) |  |  | 184 (26.67) | 133 (35.95) | 51 (15.94) |  |  |
| Retroperitoneum | 105 (10.60) | 66 (11.66) | 39 (9.18) |  |  | 65 (9.42) | 37 (10.00) | 28 (8.75) |  |  |
| Others | 115 (11.60) | 93 (16.43) | 22 (5.18) |  |  | 83 (12.03) | 68 (18.38) | 15 (4.69) |  |  |
| Tumor size, n (%) |  |  |  | χ^2^=33.11 | <0.001 |  |  |  | χ^2^=31.65 | <0.001 |
| < 5 cm | 255 (25.73) | 184 (32.51) | 71 (16.71) |  |  | 170 (24.64) | 121 (32.70) | 49 (15.31) |  |  |
| ≥ 5 cm | 467 (47.12) | 250 (44.17) | 217 (51.06) |  |  | 372 (53.91) | 188 (50.81) | 184 (57.50) |  |  |
| Unknown | 269 (27.14) | 132 (23.32) | 137 (32.24) |  |  | 148 (21.45) | 61 (16.49) | 87 (27.19) |  |  |
| Grade, n (%) |  |  |  | χ^2^=4.53 | 0.104 |  |  |  | χ^2^=1.57 | 0.457 |
| Grade I/II/III | 521 (52.57) | 298 (52.65) | 223 (52.47) |  |  | 353 (51.16) | 186 (50.27) | 167 (52.19) |  |  |
| Grade IV | 53 (5.35) | 23 (4.06) | 30 (7.06) |  |  | 45 (6.52) | 21 (5.68) | 24 (7.50) |  |  |
| Unknown | 417 (42.08) | 245 (43.29) | 172 (40.47) |  |  | 292 (42.32) | 163 (44.05) | 129 (40.31) |  |  |
| Surgery for the primary site, n (%) |  |  |  | χ^2^=11.71 | <0.001 |  |  |  | χ^2^=10.57 | 0.001 |
| No | 208 (21.01) | 97 (17.17) | 111 (26.12) |  |  | 128 (18.58) | 52 (14.09) | 76 (23.75) |  |  |
| Yes | 782 (78.99) | 468 (82.83) | 314 (73.88) |  |  | 561 (81.42) | 317 (85.91) | 244 (76.25) |  |  |
| Surgery for other regional or distant sites, n (%) |  |  |  | χ^2^=53.34 | <0.001 |  |  |  | χ^2^=28.90 | <0.001 |
| No | 872 (88.17) | 534 (94.68) | 338 (79.53) |  |  | 599 (87.06) | 344 (93.48) | 255 (79.69) |  |  |
| Yes | 117 (11.83) | 30 (5.32) | 87 (20.47) |  |  | 89 (12.94) | 24 (6.52) | 65 (20.31) |  |  |
| Chemotherapy, n (%) |  |  |  | χ^2^=243.20 | <0.001 |  |  |  | χ^2^=157.31 | <0.001 |
| No/Unknown | 281 (28.36) | 270 (47.70) | 11 (2.59) |  |  | 179 (25.94) | 168 (45.41) | 11 (3.44) |  |  |
| Yes | 710 (71.64) | 296 (52.30) | 414 (97.41) |  |  | 511 (74.06) | 202 (54.59) | 309 (96.56) |  |  |
| Radiation, n (%) |  |  |  | χ^2^=122.13 | <0.001 |  |  |  | χ^2^=85.12 | <0.001 |
| No/Unknown | 718 (72.45) | 487 (86.04) | 231 (54.35) |  |  | 480 (69.57) | 313 (84.59) | 167 (52.19) |  |  |
| Yes | 273 (27.55) | 79 (13.96) | 194 (45.65) |  |  | 210 (30.43) | 57 (15.41) | 153 (47.81) |  |  |
| Vital status, n (%) |  |  |  | χ^2^=60.17 | <0.001 |  |  |  | χ^2^=66.10 | <0.001 |
| Alive | 820 (82.74) | 514 (90.81) | 306 (72.00) |  |  | 492 (71.30) | 312 (84.32) | 180 (56.25) |  |  |
| Dead | 171 (17.26) | 52 (9.19) | 119 (28.00) |  |  | 198 (28.70) | 58 (15.68) | 140 (43.75) |  |  |
| Follow-up time, months, M (Q_1_, Q_3_) | 36.00 (36.00,36.00) | 36.00 (36.00,36.00) | 36.00 (27.00,36.00) | Z=-7.71 | <0.001 | 60.00 (37.00,60.00) | 60.00 (60.00,60.00) | 60.00 (19.00,60.00) | Z=-7.89 | <0.001 |

M, Median; Q_1_, 1st Quartile, Q_3_, 3rd Quartile.
