# Supplementary material for: Clinical characteristics and prognoses in pediatric neuroblastoma with bone or liver metastasis: data from the SEER 2010–2019
Source: BMC Pediatr. 2024 Mar 7;24:162. doi: 10.1186/s12887-024-04570-z (PMC10921780; doi:10.1186/s12887-024-04570-z)
Supplement: Supplementary file 3 — Additional file 3: Supplementary Table 3. CSS of patients with metastatic neuroblastoma and non-metastatic neuroblastoma. [file 12887_2024_4570_MOESM3_ESM.docx]

Supplementary Table 3 CSS of patients with metastatic neuroblastoma and non-metastatic neuroblastoma.

|  | Model I | | Model II | |
| --- | --- | --- | --- | --- |
| Variables | HR (95%CI) | *P* | HR (95%CI) | *P* |
| 3-year CSS |  |  |  |  |
| Metastasis |  |  |  |  |
| No | Ref |  | Ref |  |
| Yes | 3.38 (2.44-4.70) | <0.001 | 1.76 (1.21-2.56) | 0.003 |
| 5-year CSS |  |  |  |  |
| Metastasis |  |  |  |  |
| No | Ref |  | Ref |  |
| Yes | 3.29 (2.42-4.47) | <0.001 | 1.66 (1.16-2.35) | 0.005 |

Model I was adjusted for age, sex, and race;

Model II was adjusted for age, sex, race, tumor site, tumor size, grade, surgery for the primary site, surgery for other regional or distant sites, chemotherapy, and radiation.

CSS, cancer-specific survival; Ref, reference; HR, hazard ratio; CI, confidence interval.
